# Anatomical Basis for the Cardiac Interventional Electrophysiologist

**DOI:** 10.1155/2015/547364

**Published:** 2015-11-19

**Authors:** Damián Sánchez-Quintana, Manuel Doblado-Calatrava, José Angel Cabrera, Yolanda Macías, Farhood Saremi

**Affiliations:** ^1^Department of Anatomy and Cell Biology, Faculty of Medicine, University of Extremadura, 06071 Badajoz, Spain; ^2^Servicio de Cardiología, Hospital Infanta Cristina, 06006 Badajoz, Spain; ^3^Hospital Universitario Quirón-Madrid, European University of Madrid, 28223 Madrid, Spain; ^4^Department of Radiology, University of Southern California, Los Angeles, CA 90089-4019, USA

## Abstract

The establishment of radiofrequency catheter ablation techniques as the mainstay in the treatment of tachycardia has renewed new interest in cardiac anatomy. The interventional arrhythmologist has drawn attention not only to the gross anatomic details of the heart but also to architectural and histological characteristics of various cardiac regions that are relevant to the development or recurrence of tachyarrhythmias and procedural related complications of catheter ablation. In this review, therefore, we discuss some anatomic landmarks commonly used in catheter ablations including the terminal crest, sinus node region, Koch's triangle, cavotricuspid isthmus, Eustachian ridge and valve, pulmonary venous orifices, venoatrial junctions, and ventricular outflow tracts. We also discuss the anatomical features of important structures in the vicinity of the atria and pulmonary veins, such as the esophagus and phrenic nerves. This paper provides basic anatomic information to improve understanding of the mapping and ablative procedures for cardiac interventional electrophysiologists.

## 1. Introduction 

The adoption of catheter ablation techniques for treatment of tachyarrhythmias has increased interest in learning cardiac anatomy. Interventional arrhythmologists are paying more attention to study the gross morphologic and architectural features of the heart. As a result of this interest a new wave of investigation has emerged to study cardiac anatomic topics that were not completely studied in the past. Recent investigations have unraveled anatomic features, architectural aspects, and histologic details of certain components of the heart, of interest to understanding of the substrates of tachycardias and their ablation.

## 2. Right Atrium and Supraventricular Tachycardia

The right atrium (RA) is of utmost importance to electrophysiologist. It is the anatomic of major parts of the conduction system of the heart and the gateway to perform complex electrophysiological procedures that involve passing through the RA and interatrial septum. Each atrium is composed of a venous component, an appendage, body, and a vestibule. The venous component is located posterolaterally and receives the systemic venous flow, from the superior caval vein (SCV), inferior caval vein (ICV), and coronary sinus. The right atrial vestibule is a smooth muscular wall around the tricuspid orifice and supports the leaflets of the tricuspid valve. The characteristic of the vestibule is that it is surrounded by the pectinate muscles of the right atrium. The atrial chambers are separated from one another by the interatrial septum.

### 2.1. Terminal Crest (Crista Terminalis)

**Figure 1:**
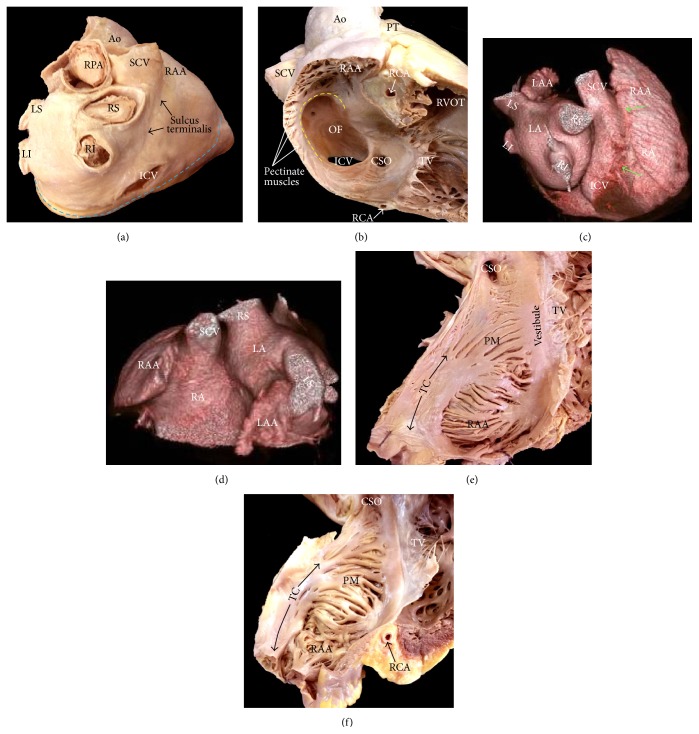
(a) The heart is viewed in attitudinally appropriate position. As can be seen, the right atrium lies anterior to its alleged left-sided counterpart. Note the arrangement of the sulcus terminalis and the atrioventricular or coronary groove (blue broken line). (b) The right atrium is shown in right anterior oblique projection. The terminal crest (yellow broken line) arches anterior to the orifice of the superior caval vein and extends toward the inferior caval vein. (b, c) Spatial relationship of the atrial structures. Right posterior (b) and superior (c) views of volume rendered CT angiographies are shown. The right atrium (RA) body (sinus venarum) is shown extending between the SCV and ICV. The right atrial appendage (RAA) is large with a wide neck compared to the left atrial appendage (LAA). Note prominent pectinate muscles of the RAA. The terminal groove (green arrows) is seen between the RAA and the venous part of the RA. The left atrium (LA) is located superior and posterior to the right atrium. (e, f) Endocardial aspects of the lateral wall of the right atrium opened. Note in (e) that the pectinate muscles have a uniform parallel alignment almost without crossovers between them. In contrast, the pectinate muscles in (f) have a nonuniform arrangement with abundant interlacing trabeculations between them. Ao = aorta, CSO = coronary sinus orifice, ICV = inferior cava vein, LA = left atrium, LAA = left atrial appendage, LI = left inferior pulmonary vein, LS = left superior pulmonary vein, OF = oval fossa, PM = pectinate muscles, PT = pulmonary trunk, RAA = right atrial appendage, RCA = right coronary artery, RI = right inferior pulmonary vein, RPA = right pulmonary artery, RS = right superior pulmonary vein, RVOT = right ventricle outflow tract, SCV = superior cava vein, TC = terminal crest, and TV = tricuspid valve.

**Figure 2:**
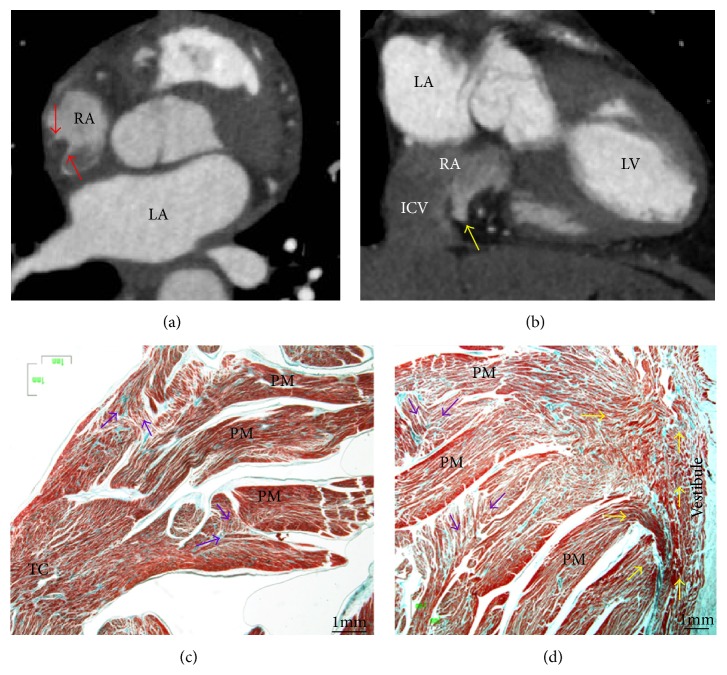
(a) Axial CT angiogram shows a prominent crista terminalis (red arrows) in the right atrium. (b) Coronal CT shows a sub-Eustachian pouch (yellow arrow) in the median aspect of the right inferior cavoatrial junction. (c, d) Frontal sections through the terminal crest at the origin and ending of the pectinate muscles. Note the irregular alignment (purple arrows) of the muscular myofibrils within the pectinate muscles and between them and the circumferentially arranged myocytes in the vestibule (yellow arrows). ICV = inferior cava vein, LA = left atrium, LV = left ventricle, PM = pectinate muscles, RA = right atrium, and TC = terminal crest.

Viewed from outside, the RA is dominated by its large, triangular appendage, which points anterosuperiorly and extends laterally (Figure 1). Usually, a fat-filled groove (sulcus terminalis, terminal groove) corresponding internally to the terminal crest (TC) or crista terminalis can be seen on the endocardial surface, demarcating the junction between appendage and venous component. The TC is an important anatomic landmark due to its close association with the sinus node region and the origin of the pectinate muscles (Figure 1). The TC is a C-shaped ridge of muscle formed by the junction of the sinus venosus and the primitive right atrium (Figure 1) [1]. The TC is a muscle bundle that rises from the RA anteromedial wall, passes in front of the SVC, and descends to the ICV to continue as an array of finer bundles that enter the region of the atrial wall recognized as the inferior isthmus or cavotricuspid isthmus (CTI) [2]. The CTI demarcates the limit between the smooth wall of the vein and the rough wall of the appendage acting as a natural barrier to the cardiac conduction system. From the lateral margin of the crest arises a series of muscle bundles known as the pectinate muscles, which fan out from the crest toward the vestibular portion (Figure 1). The TC is an important structure in several forms of atrial tachyarrhythmias and, occasionally, it is the target for radiofrequency (RF) catheter procedures [3, 4]. The myocytes in the TC are mostly aligned along the long axis of the muscle bundle, thus favoring preferential conduction. On the other hand, myocytes in the intercaval area, outside the crista terminalis, are aligned obliquely. This sudden change in orientation represents a great substrate for atrial arrhythmia [2, 5]. The nonuniform arrangement of the myocytes is probably the reason for their arrhythmogenicity. Our studies have shown morphological features relevant to electrophysiology in the TC and examined the pectinate muscles originating from the TC in 97 human necropsy heart specimens [2]. The pectinate muscles with highly trabeculated muscle fiber bundles (Figures 1 and 2) may facilitate the nonuniform spread of the excitatory impulse. The characteristic anisotropic spread of excitation from the sinus node may lead to the phenomenon of impulse “reentry” due to the timing of depolarization and refractory rates of the excitable cells [6]. This could potentially predispose patients, with significantly trabeculated pectinate muscles types, to atrial arrhythmias.

### 2.2. Sinus Node

**Figure 3:**
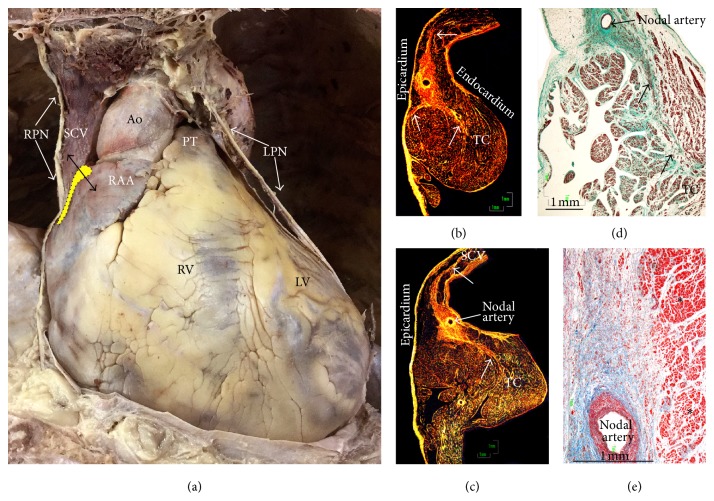
(a) Frontal view of the heart in a cadaver that has been dissected to show the course of the phrenic nerves relative to the atria and left ventricle. The anticipated location of the sinus node outlined with dots on yellow background. The double-headed arrow represents the sectioning plane used for making the sections through the sinus node and the terminal crest shown in the histological sections. (b, c) Histological sections with picrosirius red stain and polarized light showing variations in locations of the sinus node relative to the epicardial and endocardial surfaces and sizes of the terminal crest. Note nodal extensions (arrows) to superior caval vein, terminal crest, and epicardium. (d) With Masson's trichrome stain is recognizable a nodal extension to terminal crest by its fibrous matrix (green). (e) Histological section of the nodal body (Masson's trichrome stain). Note the contour of the node towards the neighboring myocardium (asterisks). Ao = aorta, LPN = left phrenic nerve, and LV = left ventricle. PT = pulmonary trunk, RAA = right atrial appendage, RPN = right phrenic nerve, RV = right ventricle, SCV = superior cava vein, TC = terminal crest, and TV = tricuspid valve.

The sinus node (SN) is the source of the cardiac impulse. In the human heart, the SN is described as an almost crescent-shaped or cigar-shaped structure usually lodged immediately subepicardially within the sulcus terminalis, lying just inferior to the crest of the atrial appendage. Our studies have shown the head and body of the node to occupy a location within the anterolateral course of the terminal groove in the vast majority of hearts [7]. Overall, the length of the node from head to tail is 13.5 mm (range 8 to 21.5 mm) [7], although the study by Matsuyama et al. [1] found nodes >30 mm long in 3/26 of their cases. In majority of hearts we studied (72%) (Figure 3), the nodal body was immediately subepicardial with a distance of 0.1–1 mm and the distance of nodal tissues from the endocardium ranged from 2.3 mm to 4.6 mm. In 28%, the distance from the endocardium was <1.5 mm. However, in hearts with terminal crests of >7 mm thickness, the nodal tail was 3.3–5.8 mm distant from the endocardial surface. The tail portion may extend inferiorly along the length of the terminal crest toward the entrance of the ICV. Fragmentation of the tail portion into separate clusters of nodal cells was found in 64% of the 47 adult hearts in our study [7].

This sinus node artery (Figure 3) is the initial branch of the right coronary artery in approximately 55% of individuals and takes its origin from the circumflex artery in the other 45%, with a few instances of lateral origin from either the right or left arteries. Histologically the SAN is composed of cells slightly smaller than normal working cells [1, 7–9]. Sinus cells are characterized by being clearer and embedded in a dense connective tissue matrix. Using semiquantitative methods, several histological studies have reported an increase in the connective tissue in the node at the expense of nodal cells with increasing age [10, 11]. Davies and Pomerance [11] noted a steady fall in the percentage surface area occupied by nodal cells in histological cross sections from age 30 to age 85 years. They commented that the node in some patients over the age of 80 had virtually no muscle yet the patients were still in sinus rhythm. In the periphery of the node, the specialized cells are mixed with those of the working myocardium. In addition, multiple radiations or extensions of the SN (Figure 3) interdigitating with the working atrial myocardium have been described [7]. These penetrate intramyocardially into the crest, epicardium, and SVC. In axial CT images the SN can be localized by locating the SA node artery along the terminal crest. Modification of the sinus node using RF has been established as a treatment for inappropriate sinus tachycardia [12]. Because of the subepicardial location of the SA node, approaching from the endocardial surface requires more RF energy to ablate the sinus node. Multidetector computed tomography (MDCT) can be used to measure the thickness of the crest and demonstrate the approximate location of the SAN artery within the nodal tissue. The centrally located nodal artery (Figure 3) may provide a cooling effect, reducing radiofrequency damage [7].

### 2.3. Koch's Triangle

**Figure 4:**
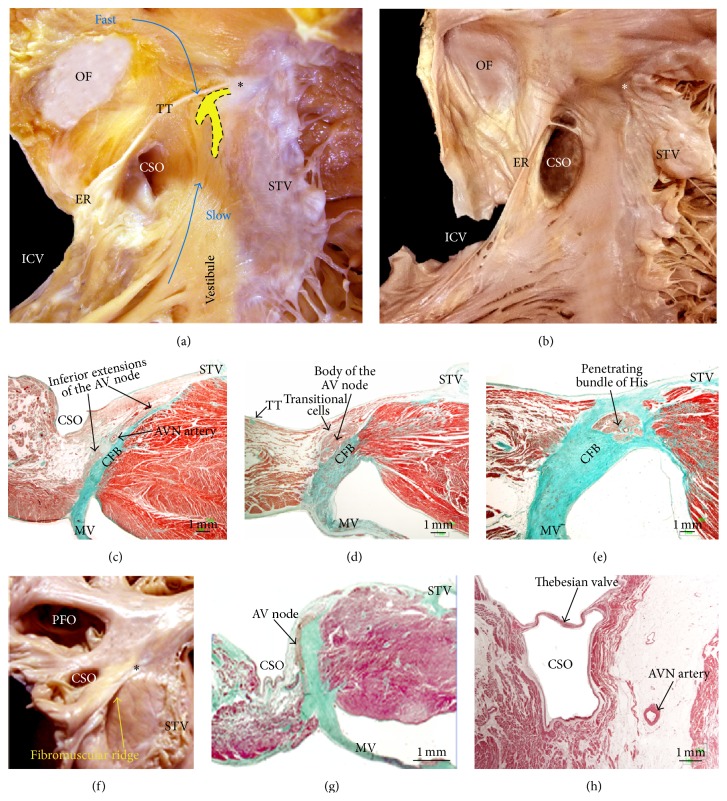
(a) Dissection in right anterior oblique view of the right atrium shows the borders of the triangle of Koch. In this view we have depicted the putative fast and slow pathways toward the AV node (dotted shape in yellow). (b) Example of small triangle of Koch with a bigger coronary sinus ostium size. (c, d, e) A series of histological sections in comparable orientation to the picture (a) are taken through the coronary sinus ostium and inferior extensions of the AV node, the body of the AV node, and the penetrating bundle of His. (f, g) Hearts with Ebstein's anomaly. Note in (f) the smaller size of the triangle of Koch and the fibromuscular ridge in relation to the normal insertion of the septal leaflet of the tricuspid valve and the dysplasia of the septal leaflet. In the sagittal histological section (g) note how in the specimen with Ebstein's anomaly the body of the AV node is at the level of coronary sinus ostium. (h) Sagittal section through the mouth of the coronary sinus showing the proximity of the AV nodal artery to the endocardium at the base of the triangle of Koch. Asterisk (*∗*) = central fibrous body, AVN artery = atrioventricular nodal artery, CSO = coronary sinus ostium, CFB = central fibrous body, ER = Eustachian ridge, ICV = inferior cava vein, MV = mitral valve, OF = oval fossa, PFO = patent foramen ovale, STV = septal leaflet of the tricuspid valve, and TT = tendon of Todaro.

Another area of significance to electrophysiologists is the triangle of Koch that delineates the location of the atrioventricular node (AVN) (Figure 4). This triangle is bordered posteriorly by a fibrous extension from the Eustachian valve called the tendon of Todaro. The anterior border is demarcated by the hinge (annulus) of the septal leaflet of the tricuspid valve. The apex of this triangle corresponds to the central fibrous body (CFB) of the heart where the His bundle penetrates. The inferior border of the triangle is the orifice of the coronary sinus (CS) together with the vestibule immediately anterior to it. The vestibular portion is the area often targeted for ablation of the slow pathway in atrioventricular nodal reentrant tachycardia (AVNRT) [13]. The so-called fast pathway corresponds to the area of musculature close to the apex of the triangle of Koch [13]. The dimensions of Koch's triangle vary from one individual to another. This variation in sizes is clinically relevant in catheter ablation procedures of this area which are largely guided by anatomic landmarks. Some reports [14] have attributed the large size of the CS ostium and a windsock morphology of the CS to increased risk factor to AVNRT (Figure 4). Because different conduction properties of the two (or more) AV pathways are key to tachycardia induction, increased distance could result in significantly different conduction properties. Although the exact reentrant circuit of AVNRT is still unknown, several hypotheses about potential inputs and courses of the activation wave-front are under discussion [15]. The lowest rate of complete AV nodal block is reported for the ablation attempt of the so-called slow pathway, whereas fast pathway modification has been abandoned because it carries a high risk of complete AV block.

The body of the AVN is found near the apex of the triangle and the His bundle penetrates the CFB at the apex. The penetrating His bundle can readily be distinguished from the compact node at the point where the conduction axis itself becomes completely surrounded by tissues of the CFB (Figure 4) and conducts electrical pulses to the ventricles [9]. The AVN consists of a compact portion and an area of transitional cells. The compact AV portion lies over the CFB (Figure 4). The size of the transitional cells is intermediate between those of the AV node and the atrial working cells. They are surrounded by a greater quantity of connective cells than that covering the working cells, but they are not insulated from the adjacent myocardium. Instead, they form a kind of bridge between the working and nodal myocardium and collect electrical information from the atrial walls, transmitting it to the AV node [9]. Toward the inferior portion of the triangle, there are extensions of nodal tissues that extend inferiorly to the right and left toward the tricuspid and mitral valves, respectively [16, 17]. These nodal extensions have been implicated in slow pathway conduction. Because this area also contains the zone of transitional cells that feeds into the compact AV node, this too may have a role in slow pathway conduction. Multicomponent atrial electrograms and “slow pathway potentials” are helpful in identifying target sites for radiofrequency catheter ablation of the slow pathway in patients with AVNRT. Low amplitude with fractionated electrograms in the base of the triangle of Koch near the coronary sinus and anterior aspects of the tricuspid annulus is now the key guidance to identify the slow pathway area [18, 19]. In some circumstances, the AV node components can be displaced, increasing the risk of total AV block when attempting slow pathway ablation. AV node displacement occurs in Ebstein malformation [20] and persistent left SVC with a grossly enlarged CS ostium. In both abnormalities the size of triangle of Koch is reduced (Figure 4), resulting in shorter distance between the compact AV node and the CS ostium.

The inferior pyramidal space is the extracardiac fibroadipose tissue wedging between the atrial and ventricular musculatures [21]. Important structures including the orifice of the coronary sinus, atrioventricular node, atrioventricular nodal artery, muscular atrioventricular sandwich, and aortic and atrioventricular valvar attachments are located around or inside the inferior pyramidal space. These relationships can be easily shown with CT angiography of the heart [22]. The AVN artery originates from the apex of the U-turn of the distal right coronary artery (RCA) and penetrates into inferior pyramidal space at the level of crux of the heart in 80–87% of patients [23]. In the remaining percentage of patients, it originates from the terminal portion of the left circumflex artery (LCx) (8–13%) or, uncommonly, from both the RCA and the LCx (2–10%). The artery provides branches to the posterior interventricular septum, interatrial septum, AVN, and penetrating bundle of His [23]. In some patients, at the level of Koch's triangle the AVN artery runs just beneath the endocardium near the ostium of the CS and septal isthmus (Figure 4). This may explain the possible risk of AVN artery coagulation during radiofrequency ablation in the slow pathway region, although a complete AV block is commonly a direct result of tissue injury to the AVN. In a study of cadaveric hearts we have found that the mean distance of the artery to the endocardial surface at the base of Koch's triangle was 3.5 ± 1.5 mm [23].

### 2.4. Right Atrium Isthmus-Dependent Atrial Flutter

**Figure 5:**
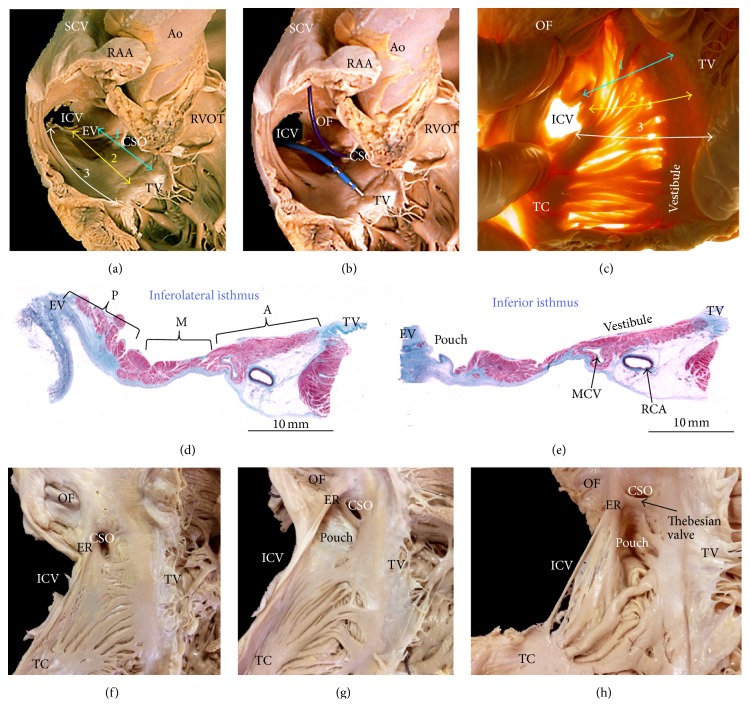
(a) The region of the cavotricuspid isthmus in simulated right anterior oblique (RAO) view and the paraseptal, inferior, and inferolateral isthmuses are marked 1, 2, and 3, respectively. (b) Opened right atrium in simulated RAO view showing the position of the ablation catheter at the site of inferior or central isthmus (marked with number 2) of application of radiofrequency. (c) This atrial view shows the cavotricuspid isthmus with transillumination. The lines mark (1) the paraseptal isthmus, (2) the inferior isthmus, and (3) the inferolateral isthmus. Note the smooth vestibule immediately proximal to the tricuspid valve and the pectinate muscles in the posterior regions. (d, e) This series of histological sections through inferolateral (d) and inferior (e) isthmuses from a heart with dominance of right coronary artery. Note in (d) the prominent and fibromuscular Eustachian valve in the posterior sector or P, thin myocardium in middle sector or M, and thicker myocardium in the anterior sector (vestibule) or A. In (e), histologic section shows a pouch of the sub-Eustachian recess. Note the lesser transmural thickness in this area. The right coronary artery is in the epicardial fat related to the smooth vestibule. (f, g, h) These hearts show variations in morphology of the Thebesian valve guarding the coronary sinus orifice, the sub-Eustachian pouch, and Eustachian ridge. Ao = aorta, CSO = coronary sinus orifice, EV = Eustachian valve, ER = Eustachian ridge, ICV = inferior cava vein, MCV = minor coronary vein, OF = oval fossa, PT = pulmonary trunk, RAA = right atrial appendage, RCA = right coronary artery, RVOT = right ventricle outflow tract, SCV = superior cava vein, TC = terminal crest, and TV = tricuspid valve.

The cavotricuspid isthmus (CTI) in the lower part of the right atrium between the ICV and the tricuspid valve is considered crucial in producing a conduction delay and, hence, favoring the perpetuation of a reentrant circuit. Atrial flutter (AFL) can be categorized as being dependent on the CTI (or typical) and non-isthmus-dependent (or atypical). The commonest type of AFL is isthmus-dependent AFL, which is a stable macroreentrant circuit that has its basis in the anatomical structure of the right atrium. The wave-fronts progress around the tricuspid annulus in a counterclockwise direction, resulting in the typical sawtooth-like P-wave morphology. The target for ablation is at the level of the isthmus of right atrium wall between the orifices of the inferior vena cava and the tricuspid valve [24]. The flutter isthmus is bordered anteriorly by the annulus of the tricuspid valve and posteriorly by the Eustachian valve (Figure 5). Autopsies, angiographic, and echocardiographic studies have shown that the anatomy of flutter isthmus is highly variable [25–27]: patients with a short and straight CTI require fewer radiofrequency ablation applications and shorter X-ray exposure. Obstacles such as a large Eustachian ridge/valve (ER) or a deep sub-Eustachian sinus may also lead to longer and more difficult ablation sessions (Figures 2 and 4). In our study of cadaveric hearts we divided the CTI into three parallel levels [25, 28]. With the heart in attitudinal orientation, we identified and measured the lengths of three levels of the isthmus: paraseptal (24 ± 4 mm), inferior (19 ± 4 mm), and inferolateral (30 ± 3 mm). The paraseptal isthmus forms the base of Koch's triangle (Figure 5) and is commonly referred to as septal isthmus. The septal isthmus is the shortest of the three isthmuses but has the thickest wall ranging from 2 to 7 mm on heart specimens and it is closest to the AV node, particularly the inferior nodal extensions. The inferior isthmus, also known as the “central isthmus” owing to its location between the other two isthmuses (6 o'clock on LAO projection), represents the optimal target for ablation since this is the thinnest site between the ICV orifice and the tricuspid valve annulus. It is also shown that most diameters are larger in patients with chronic atrial flutter (larger RA) compared to paroxysmal atrial flutter or control groups [29]. In cadaver hearts, all three isthmuses have in common a smooth anterior zone being the right atrium vestibule. The distance between the right coronary artery and endocardial surface of the vestibule ranges from 2 to 11 mm. By contrast, the posterior zone is composed of mainly fibrous and fatty tissue as it joins the Eustachian valve (Figure 5). Between the anterior and posterior zones, the morphology and thickness of the isthmus are highly variable. In approximately 20% of patients there is a pouch-like recess known has the sub-Eustachian (sub-Thebesian) sinus or recess in the inferior isthmus (Figures 2 and 5) which can cause considerable difficulty in achieving a complete line of block in this isthmus and is the reason for the preference of some electrophysiologists to ablate an alternative right atrium isthmus line instead.

Anatomical studies with CT scan using image data obtained during different cardiac phases have shown that the CTI is longest at all three parallel levels during midventricular systole [30]. In this phase the central isthmus length is greater than 30 mm in 32% of patients. The CTI varies in size during the cardiac cycle, becoming deeper with atrial contraction. The measurements have also shown a total of 40% variation in length during a single cardiac cycle. In around 28% of the population, a “hook-shaped” morphology is seen, showing a concave or pouch-like segment posteriorly (at the ICV side) and a flat vestibular part anteriorly.

### 2.5. The Eustachian Valve or Ridge

For the electrophysiologist entering the right atrium via the inferior route, the first structure encountered is the Eustachian valve (EV) guarding the orifice of the ICV. Normally, it is a thin, insignificant, crescentic flap. Occasionally, the flap is large and may impede access to the most posterior part of the isthmus. The free border of the EV continues as the tendon of Todaro that runs in the musculature of the Eustachian ridge (Figure 5). It has been demonstrated that, in patients with a large Eustachian ridge/valve, the paraseptal isthmus block can be obtained only after the complete ablation of the enlarged Eustachian ridge [31]. In our study of cadaveric hearts it was demonstrated that 26% of their heart specimens had a thickened Eustachian ridge, with a mean thickness of 3.2 ± 0.8 mm [25]. A thick Eustachian ridge greater than 4 mm is seen in 24% of the normal population studied with CT scans [30]. An angiographic study carried out by Heidbüchel et al. [26] revealed an enlarged Eustachian valve in 24% of patients, with a consequent increase in the number of ablation pulse applications required for the achievement of a successful block. In approximately 2% of the population, the EV has a fishnet appearance of varying size. This is known as a Chiari network and when electrophysiologists encounter it, they should be aware of the potential risk of the catheter being caught by the network [32].

### 2.6. The Sub-Eustachian Sinus (Sinus of Keith, Sub-Thebesian Recess)

The sub-Eustachian recess is an extension of a pouch-like isthmus under the orifice of the coronary sinus (Figure 5). Attitudinally appropriate nomenclature for this anatomic variant is sub-Thebesian recess [33]. The presence of a large sub-Eustachian recess or deep pouches is associated with significantly more RF applications as compared with straight isthmus [34]. Local radiofrequency delivery may be impaired by this structure because an area of limited blood flow results in delayed catheter tip cooling. In one angiographic study of the CTI, this pouch was observed in 47% of patients and had a mean depth of 4.3 ± 2.1 mm (1.5 to 9.4) [26]. CT scans in normal population have identified a deep recess combined with a pouch-like (greater than 5 mm in 45% of patients) central isthmus in 45% of mid-diastolic phase images [30]. This finding would be useful in preprocedural planning, where the presence of a large pouch would dictate a central approach to the ablation.

## 3. The Atrial Septum and Transseptal Access

The anatomy of the interatrial septum (IS) is particularly important for interventional cardiologists. The fact that the left atrium (LA) is posterior and superior to the RA determines the orientation of the atrial septum. Generally, the septal plane is at an angle to the median sagittal plane when viewed from the front of the chest, and a right anterior oblique projection will view the septal plane more or less “en face.”

**Figure 6:**
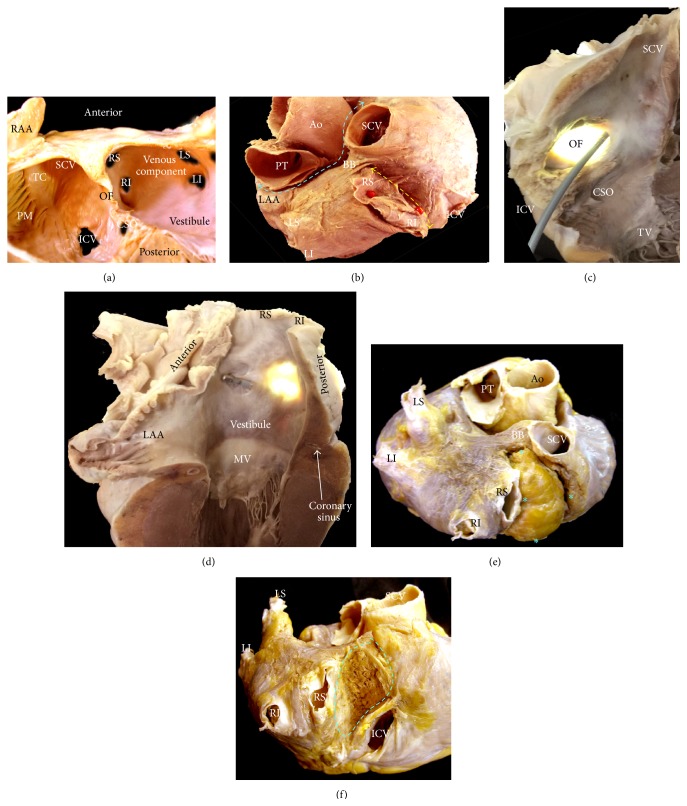
(a) Dissection through the atria with parts of the anterior walls removed and viewed from a left anterior perspective to show the interatrial septum and components of the left atrium and its relatively smooth endocardial surface. (b) This superior view of the heart showing the transverse sinus (blue broken line), the posterior interatrial groove (yellow broken line), and Bachmann's bundle crossing the interatrial grove. (c, d) Longitudinal sections through the venous component of the right atrium showing by transillumination in (c) the flap valve of the oval fossa and the muscular rim that surrounds it on the right atrial aspect. In this heart there is probe patency of the oval fossa, leaving a gap in its anterosuperior aspect. The gap can allow a catheter to be slipped between the rim and the valve (c) to enter the left atrium (d). Note in (d) by transillumination the location of the oval fossa in the left side of the septum. (e, f) Lipomatous hypertrophy of the interatrial septum. In (e) blue asterisks indicate the total volume of fat tissue and its anatomical distribution within the interatrial septum. Note in (f) that the tumor was excised of the interatrial septum and its extension is highlighted by a blue broken line. Ao = aorta, BB = Bachmann's bundle, CSO = coronary sinus orifice, ICV = inferior cava vein, LAA = left atrial appendage, LI = left inferior pulmonary vein, LS = left superior pulmonary vein, MV = mitral valve, OF = oval fossa, PM = pectinate muscles, PT = pulmonary trunk, RAA = right atrial appendage, RI = right inferior pulmonary vein, RS = right superior pulmonary vein, SCV = superior cava vein, TC = terminal crest, and TV = tricuspid valve.

On heart specimens, the septal aspect of the RA gives an erroneous impression of there being an extensive atrial septum. The true septum, as defined by the area that can be excised without exiting the heart, is limited to the area marked by the valve of the oval fossa (the embryonic septum primum) and the anterior buttress of the atrial septum that is the true “secondary septum” [35]. The superior rim of the fossa is an infolding and is not strictly “septal.” Spatial orientation of the anatomic components of the interatrial septum is best shown by CT angiography [36–38]. After birth, the embryonic shunt is eliminated when the valve of the fossa closes against the muscular rim. The rim is an infolding of the atrial wall. The superior and posterior parts of the rim represent the infolding between the SVC and the right pulmonary veins (Figure 6). It lies behind the transverse pericardial sinus and the aortic root. The anterior and inferior parts of the rim are separated from ventricles by the fat-filled inferior pyramidal space, through which the AVN artery passes. The left atrial aspect of the atrial septum lacks the crater-like feature of the right side because it is the fossa valve that overlaps the fossa rim on the right side (Figure 6). The valve itself is usually thin (1 to 3 mm) and fibromuscular and comprises a bilaminar arrangement of myocardial strands aligned in different directions. In about one-third of the normal population, there is probe patency of the oval fossa (Figure 6), even though on the left atrial side the valve is large enough to overlap the rim. This is because the adhesion of the valve to the rim is incomplete, leaving a gap usually in the anterosuperior margin corresponding to a C-shaped mark in the left atrial side. The gap can allow a catheter to be slipped between the rim and the valve to enter the left atrium (Figure 6).

Two main anatomic variants of the interatrial septum are lipomatous hypertrophy of the septum (Figure 6) and large septal aneurysm (the valve tends to be thinner and stretchier), and both can be clearly demonstrated by two-dimensional transesophageal echocardiography (TTE) and by CT or cardiac magnetic resonance (CMR), although three-dimensional TTE and CT-derived images are anatomically the most accurate [36–38]. Lipomatous hypertrophy of the septum is accumulation of fat in the interatrial groove not in the true septum [37]. Moreover, increased size of the atrial chambers (e.g., increased age or body mass), severe kyphoscoliosis, marked left ventricular hypertrophy, or an enlarged aorta can affect the orientation of the septal plane and may result in displacement of the oval fossa [39]. The risk of perforating the heart is higher in such cases when attempting to circumvent the barrier by puncturing the peripheral of the true septum. Therefore, right atrium morphology should be assessed before any electrophysiological or interventional procedure that involves a transseptal approach. CT and CMR are able to adequately show the atrial septal anatomy. Because the majority of these techniques require the visualization of the left atrial appendage in search of a thrombus, transesophageal echocardiography is the method of choice. Although two-dimensional and three-dimensional TEE are the most cost effective methods, three-dimensional TEE enables an “en face” view of the anatomy of the atrial septum, even during intervention, which is why this method is preferable when available [40].

## 4. The Left Atrium and Pulmonary Veins Relevant to Atrial Fibrillation Ablation

Because the left atrium (LA) is the main target of catheter ablation in patients with atrial fibrillation (AF), we review the gross morphological features of this chamber, the anatomy of pulmonary veins (as major target sites), and their relations with collateral extracardiac structures (e.g., phrenic nerves, the esophagus) which is key to successful and, more importantly, safe ablation.

### 4.1. The Left Atrium

**Figure 7:**
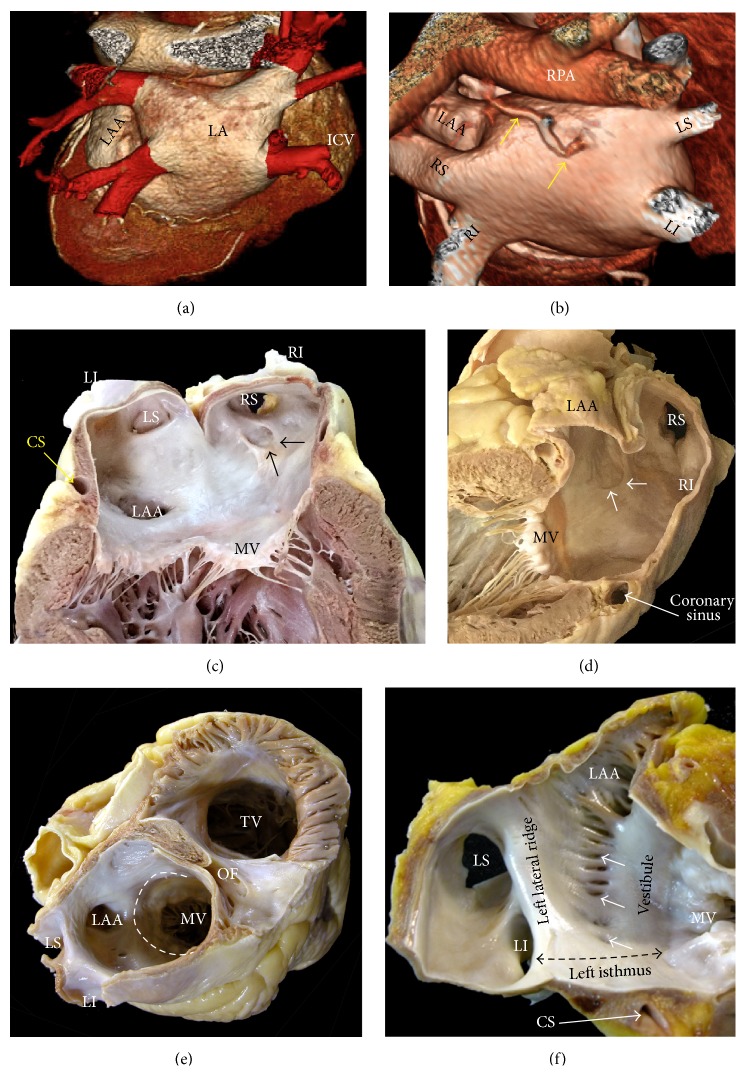
(a, b) Spatial relationship of the left atrial structures. Posterosuperior view of volume rendered CT angiographies is shown. The left atrium is located in superior and posterior aspect of the heart. (a) Normal arrangement of the pulmonary veins (in red) is shown. The left atrial appendage (LAA) is shown in yellow. (b) Again shown are normal pulmonary veins. A supernumerary pulmonary vein (yellow arrows) is shown arising from the superior wall of the left atrium. The supernumerary pulmonary vein usually drains a portion of the right upper lung and inserts directly into the left atrium body or at the junction of the right superior pulmonary vein (RS) and the left atrium. (c) Longitudinal section through the left atrium and left ventricle, showing the smooth endocardial aspect of the left atrium. The black arrows indicate the crescentic edge of the oval fossa valve. (d) Longitudinal section through the pulmonary venous component showing the orifices of the right pulmonary veins. Note the close relation of the right veins orifices with the atrial septum (white arrows). (e) Short-axis section across the atrial chambers to show the atrioventricular valves, the vestibule of the left atrium (dotted line), and the nonuniform thickness of the left atrial wall. (f) Endocardial visualization of the left posterolateral wall showing a prominent left lateral ridge and extra pectinate muscle trabeculations (arrows) extending inferiorly from the appendage to the vestibule of the mitral valve. Double-headed black broken line is showing the left atrial isthmus. CS = coronary sinus, ICV = inferior cava vein, LA = left atrium, LAA = left atrial appendage, LI = left inferior pulmonary vein, LS = left superior pulmonary vein, MV = mitral valve, OF = oval fossa, RPA = right pulmonary artery, RI = right inferior pulmonary vein, RS = right superior pulmonary vein, and TV = tricuspid valve.

**Figure 8:**
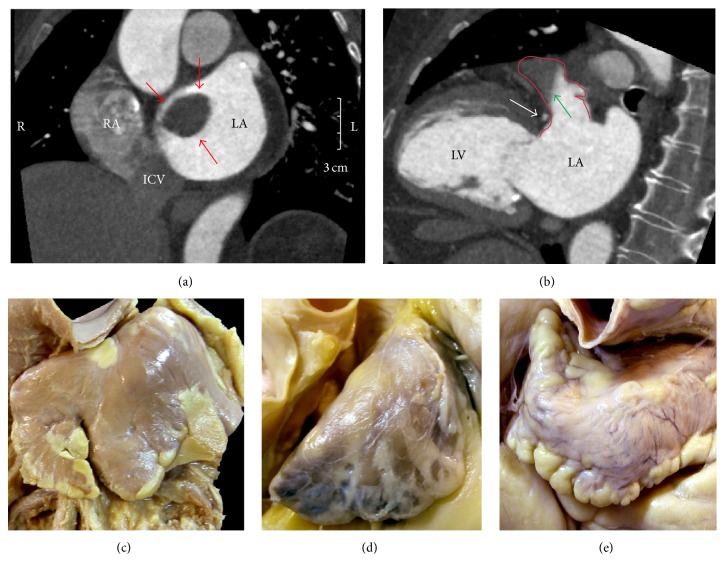
(a) Short-axis view of CT angiography showing a large clot (red arrows) in the left atrium attaching to the interatrial septum in a patient with atrial fibrillation. (b) Long axis view of the left heart showing the left atrial appendage (bordered in red) containing fluid-fluid level of unmixed contrast (green arrow) due to incomplete contraction of the left atrial appendage in this patient with atrial fibrillation. Note close anatomic course of the left circumflex artery (white arrow) near the neck of the left atrial appendage. (c, d, e) Images demonstrating significant interindividual variation in left atrial appendage morphology ((c) chicken wing; (d) cauliflower; and (e) windsock). ICV = inferior cava vein, LA = left atrium, LAA = left atrial appendage, LV = left ventricle, and RA = right atrium.

As with the RA, the LA possesses a venous component, a vestibule, a body, and an appendage. The pulmonary venous component, with the venous orifices at each corner, is found posteriorly. The vestibular component surrounds the mitral orifice [41]. The major part of the LA, including the pulmonary venous component, vestibule, and septal component, is smooth walled (Figures 6 and 7). The left atrial appendage (LAA) is a small finger cul-de-sac in human hearts where thrombi may form; it is derived from the primitive atrium and has a rough, trabeculated surface (Figures 6, 7, and 8). Viewed from the frontal aspect of the chest, the LA is the most posteriorly situated of the cardiac chambers. The pulmonary veins (PVs) enter the posterior part of the LA with the left veins located more superiorly than the right veins. The anterior wall of the LA is situated behind the transverse pericardial sinus. A small area of the anterior wall immediately inferior to Bachmann's bundle (interauricular muscle band) can be very thin (less than 2 mm) and is at risk of perforating into the transverse pericardial sinus. The esophagus and descending thoracic aorta are immediately behind the pericardium of the posterior wall of the LA. It tends to become thinner toward the orifices of the PVs (Figures 6 and 7). The coronary sinus with its continuation, the great cardiac vein, tracks along the outside of the posteroinferior wall of the LA (vestibule) (Figure 7). The coronary sinus is wrapped by its own myocardial sleeve, which covers a length of approximately 2.5 to 5 cm with increasing thickness toward the coronary sinus ostium. Often, there is continuity between the musculature of the venous sleeve and the posterior atrial wall. The superior wall, or roof, is the thickest wall of the LA (Figure 7), measuring 3.5 to 6.5 mm, and related to the bifurcation of the pulmonary trunk and right pulmonary artery.

**Figure 9:**
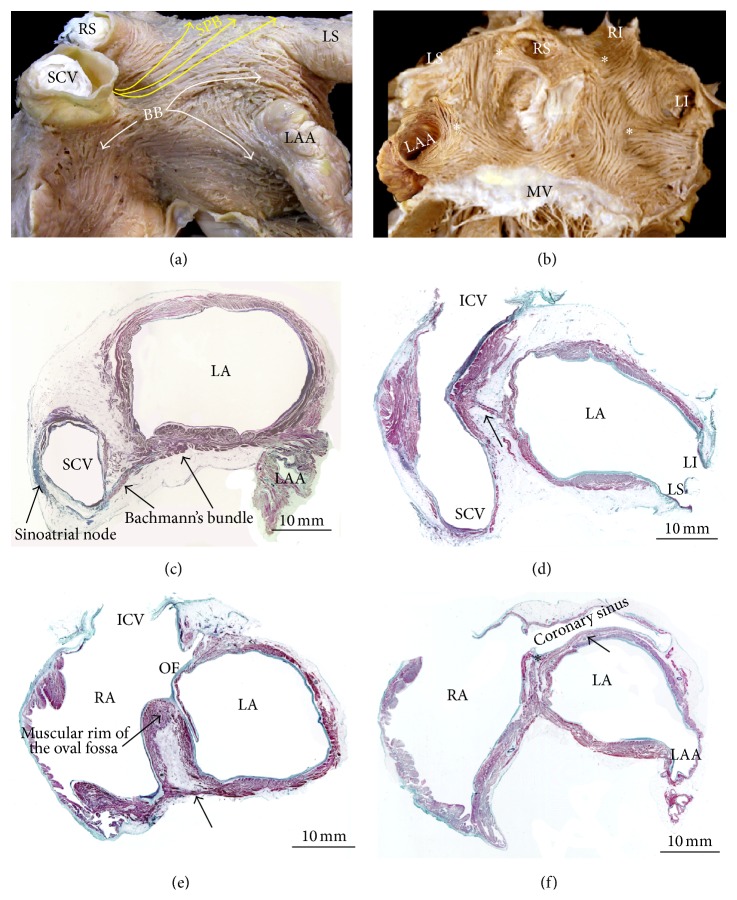
(a) Dissection to show Bachmann's bundle, crossing the anterior interatrial groove and branching toward the left atrial appendage (white lines), and the septopulmonary bundle, which arises from the interatrial groove underneath Bachmann's bundle, fanning out to line the pulmonary veins and to pass longitudinally over the dome (yellow lines). (b) Dissection of the subendocardium showing the abrupt changes of subendocardial fiber orientation (asterisks) of the septoatrial bundle at the level of the posterior wall of the left atrium, left atrial appendage, and orifices of the pulmonary veins. (c) Cross-histological section (Masson's trichrome stain) shows Bachmann's bundle and its rightward extension toward the sinus node. (d) Cross-histological section showing a muscular bridge (arrow) across the anterior interatrial groove connecting the right atrium to the left atrium. (e) Cross-histological section showing a muscular bridge below Bachmann's bundle crossing the anterior interatrial groove (arrow). (f) Cross-histological section (Masson trichrome stain) through the CS demonstrates the coronary sinus and left atrial muscle connection (asterisk) at the distal end of the coronary sinus. Often there is continuity between musculature of the coronary sinus sleeve and left atrial wall (arrow). BB = Bachmann's bundle, ICV = inferior cava vein, LA = left atrium, LAA = left atrial appendage, LI = left inferior pulmonary vein, LS = left superior pulmonary vein, MV = mitral valve, OF = oval fossa, RA = right atrium, RI = right inferior pulmonary vein, RS = right superior pulmonary vein, SCV = superior cava vein, and SPB = septopulmonary bundle.

The walls of the left atrial body are composed of several overlapping layers of differently aligned myocardial strands, and there are marked regional variations in muscular thickness [41]. In the anterosuperior wall of the LA, Bachmann's bundle (Figure 9) is the most superficial of the myocardial layers and composed of nearly parallel alignment of myocardial strands, which accounts for its role as the prevalent interatrial conduction pathway for propagation of the sinus node impulse to the anterior left atrial wall. Rightward, this bundle, after crossing the interatrial groove, bifurcates to embrace the right atrial appendage extending to the sinus node and terminal crest. Leftward, Bachmann's bundle branches to pass around the neck of the left atrial appendage, joining to continue into the musculature of the lateral and posteroinferior atrial walls [41].

The epicardial fibers of the superior wall are composed of longitudinal or oblique fibers from the septopulmonary bundle [41] (Figure 9) that arise from the anterosuperior septal rim of the oval fossa beneath the Bachmann bundle. As they ascend the roof, they fan out to pass in front of, between, and behind the right and left PVs and the myocardial sleeves that surround the venous orifices. In the subendocardium, the myocytes of the septoatrial bundle encircle the ostium of the left atrial appendage [41] (Figure 9) and continue into the pectinate muscles within the appendage, the venoatrial junctions, and the myocardial sleeves of the PVs. The interpulmonary area or carina may show abrupt changes in alignment of the cardiomyocytes. It is important to remind that neither Bachmann's bundle nor the septoatrial bundle are insulated from the remainder of the left atrial cardiomyocytes. It is the preferential alignment of the cardiomyocytes that permits the recognition of these “bands” or “bundles.”

In addition to Bachmann's bundle, there are other interatrial muscular connections of varying thicknesses and widths that cross the interatrial groove (Figure 9) and connect the muscular sleeves of the right PVs to the right atrium, the SCV to the LA, or the coronary sinus and vein of Marshall to the LA, and the intercaval area of the right atrium to the muscular sleeves of the coronary sinus, providing the potential for inferior breakthrough of the sinus impulse.

The venous component of the LA receives the PVs, whereas the vestibular components surround the opening to the mitral valve. There are no endocardial landmarks to separate the vestibule from the pulmonary venous components (Figures 6 and 7). The left atrial appendage tends to have a tubular shape with one or several bends resembling a little finger (Figure 8). On the endocardial aspect, the ostium of the LAA is narrow, oval in shape, and fairly well defined. There is considerable variability in the shape and size of the appendage due to its lobes and branches (Figure 8). A histopathological study reported the appendages to be larger and associated with more fibroelastosis in patients with AF than in those without arrhythmia [42]. The tip of the appendage can be directed anteriorly overlying the pulmonary trunk, superiorly behind the arterial pedicle, or posteriorly. When the tip is directed anteriorly, the body of the appendage usually also overlies the main stem of the left coronary artery, the great cardiac vein, and the left circumflex artery (Figure 8).

Unlike the RA, the LA lacks a terminal crest. The most prominent ridge in the LA is the left lateral ridge between the orifices of the left PVs and the ostium of the LAA [43] (Figure 7). This structure is an infolding of the lateral atrial wall protruding into the endocardial LA surface as a prominent ridge. Our anatomical study confirmed a narrow ridge of <5 mm in majority of specimens, and the ridge had thicker myocardium superiorly rather than inferiorly [43]. No significant differences were found between hearts with and without structural heart disease. Stability and contact of the ablation catheter may become challenging when constructing pulmonary vein isolation lines in cases with narrowed ridges. The epicardial side of the ridge contains the oblique vein of Marshall with its accompanying autonomic nerves [43], and, occasionally, the artery supplying the sinus node also runs in the ridge. The oblique vein (of Marshall) passes from being superiorly in relationship with the left PVs to descend along the left lateral ridge to join the coronary sinus and can be the source of atrial fibrillation [44]. This vein is short (2-3 cm), and its superior part can be obliterated by fibrosis. It presents a complete fibrosis or obliteration in the form of a cord or ligament in 5–12% of cases [45] and if adequately wide, this vein may be utilized for ablating the left atrial wall. Sometimes, It remains patent as an isolated malformation, persistent left SVC (in 0.3% of the normal population) draining into the coronary sinus, which has an enlarged ostium. In some hearts, one-third of the normal population, the endocardial aspect of the left atrial body around the ostium of the LAA presents between the left lateral ridge and the vestibule of the mitral valve a complicated network of fine extra-appendicular pectinate muscles with small pits and troughs where the wall becomes paper thin [43] (Figure 7). So electrophysiologists, to perform ablation in this region, should be mindful of the potentially thin atrial wall and crevices that may entrap catheters. These may be encountered when constructing ablation lines along the so-called left atrial isthmus to link the orifice of the left inferior pulmonary vein to the mitral annulus (Figure 7). The left atrial isthmus varies in length from 17 to 51 mm [46], and its average myocardial thickness is approximately 4 mm at the midportion. In most adults hearts the coronary sinus course outside of the vestibule 6 to 10 mm proximal to the level of the mitral annulus. MDCT can be helpful in assessing the boundaries of this area including the exact location of the mitral valve, coronary sinus, circumflex artery, and great cardiac vein and their anatomic variants.

### 4.2. The Pulmonary Veins

**Figure 10:**
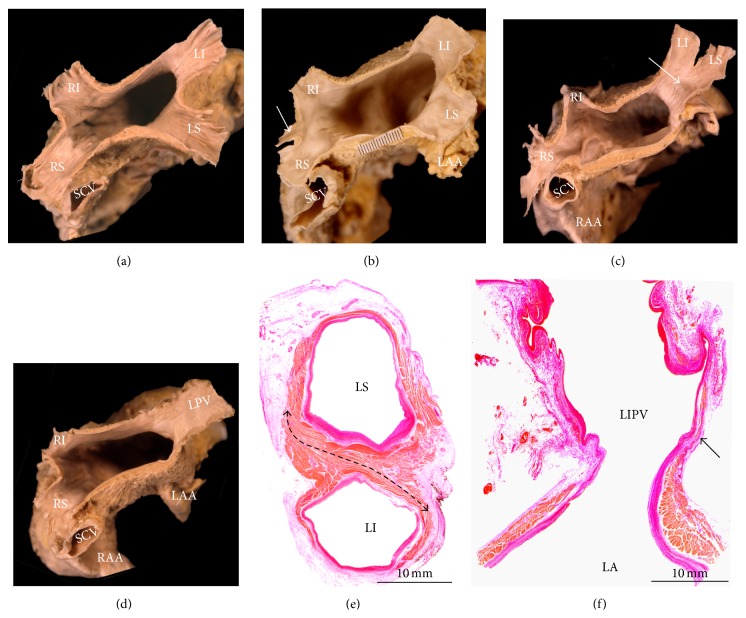
(a–d) Transverse section of four heart specimens with the roof of the left atrium removed and viewed from above to show the entrance of the pulmonary veins. Note, in (a), the arrangement of four individualized endings of the pulmonary veins into the left atrium. In (b), a separate right middle pulmonary vein (arrow) can be seen, which drains the middle lobe of the lung. (c) showed a conjoined ostia (arrow), a common variant seen in up to 25% of cases, on the left side. In (d) a single left pulmonary vein can be seen. (e) Cross-histological section of the left PVs stained with elastic van Gieson. Note the interpulmonary myocardial connections (double-headed black broken line) between the left superior and inferior veins. Also note a nonuniform in circumferential thickness of the myocardial sleeves in left pulmonary veins. (f) Longitudinal histological section stained with elastic van Gieson showing the thicker atrial wall becoming thinner at the entrance of the left inferior pulmonary vein to form the muscular sleeve, which tapers toward the lung. Note the presence of gaps of connective tissue bridges between the myocytes (arrow). LA = left atrium, LAA = left atrial appendage, LI = left inferior pulmonary vein, LPV = left pulmonary vein, LS = left superior pulmonary vein, RAA = right atrial appendage, RI = right inferior pulmonary vein, RS = right superior pulmonary vein, and SCV = superior cava vein.

In formalin fixed adult cadaveric hearts normal pulmonary vein (PV) anatomy consists of two right-sided and two left-sided PVs with separate ostia [47] (Figures 7 and 10), but there are many variables. In anatomical studies with MDCT the PV ostia usually appear ellipsoid with a longer superiorinferior dimension. The orifices of the right PVs are directly adjacent to the plane of the atrial septum (Figure 7). The right superior PV is located close to the SVC or RA, and the right inferior PV projects horizontally. The left superior PV is close to the LAA and the left inferior PV courses near the descending aorta. The veins are larger in AF versus non-AF patients, men versus women, and persistent versus paroxysmal arterial fibrillation patterns. The PV trunk is defined as the distance from the ostium to the first-order branch. The superior pulmonary vein ostia are larger (19-20 mm) than the inferior pulmonary vein ostia (16-17 mm) [48]. The superior pulmonary veins tend to have a longer trunk (21.6 ± 7.5 mm) than the inferior PVs (14.0 ± 6.2 mm) [48]. It is important to note the ostial diameters of each vein and the length to the first-order branch. These diameters influence the selection of the circular catheter size used. Common anomalies include a conjoined (common) left or right pulmonary vein in 25% of individuals [41]. A conjoined PV is seen more frequently on the left than on the right side (Figure 10) [49]. Supernumerary veins are also frequent. The most common one is a separate right middle pulmonary vein, which drains the middle lobe of the lung (Figures 7 and 10). One or two middle lobe vein ostia can be seen in 26% of patients [42]. The ostial diameter of the right middle pulmonary vein is smaller than that of the other veins (mean, 9.9 ± 1.9 mm). The ectopic focus originating from the right middle PV could initiate AF, which is cured by catheter ablation of the right middle PV. In some patients, there is a supernumerary PV that shows an aberrant insertion, with a perpendicular position in relation to the LA posterior wall. The supernumerary branch usually drains the upper lobe of the right lung and characteristically passes behind the bronchus intermedius. The absence of one PV requires careful examination of the whole intrathoracic venous system since it may be associated with partial anomalous venous return. The caliber of the PVs gradually increases as they approach the left atrium. However, the caliber of the left inferior pulmonary vein may decrease as it enters the LA and this should not be mistaken for stenosis [50].

The musculature of the left atrial wall extends to varying lengths over the outer surface of the venous wall, with the longest sleeves along the upper veins [51]. The sleeves are thicker and surround the veins at the venoatrial junctions, whereas the musculature is usually thinner and tapers irregularly as the veins are traced toward the lung hilum (Figure 10). At the venoatrial junction the myocytes cross the interpulmonary isthmus or carina at the subepicardium and the subendocardium aspects connect the walls of adjacent PVs (Figure 10). The peripheral margins of the sleeves are associated with increasing fibrosis and degenerative changes of the myocytes (Figure 10). The arrangement of the myocyte bundles within the sleeves there appeared to be a mesh-like arrangement of muscle fascicles, made up of circular-orientated bundles that interconnected with bundles that ran in longitudinal or obliquely orientation [51]. Experimental studies have shown that this complex architecture may facilitate microentry and arrhythmias associated with ectopic activity. Several studies have been designed to investigate and to compare the pathology of the PVs in patients with and without AF [52–54]. Although patients with AF have a higher number of pathological alterations (e.g., the patients with AF had significantly more marked fibrosis than those in sinus rhythm), anatomical findings are similar. Other candidates for substrates of automaticity have been suggested also. These include histologically specialized conduction cells, particularly node-like cells, and interstitial Cajal cells [55, 56]. The areas of the venoatrial junctions and adjoining PVs are also highly innervated by ganglionated nerves originating from the cardiac neural plexus. The epicardial subplexuses located in the atrial fat pads send abundant intrinsic nerve extensions onto these areas and penetrate into the atrial and venous wall [57].

### 4.3. Relationship between the Left Atrium and Neighborhood Structures

**Figure 11:**
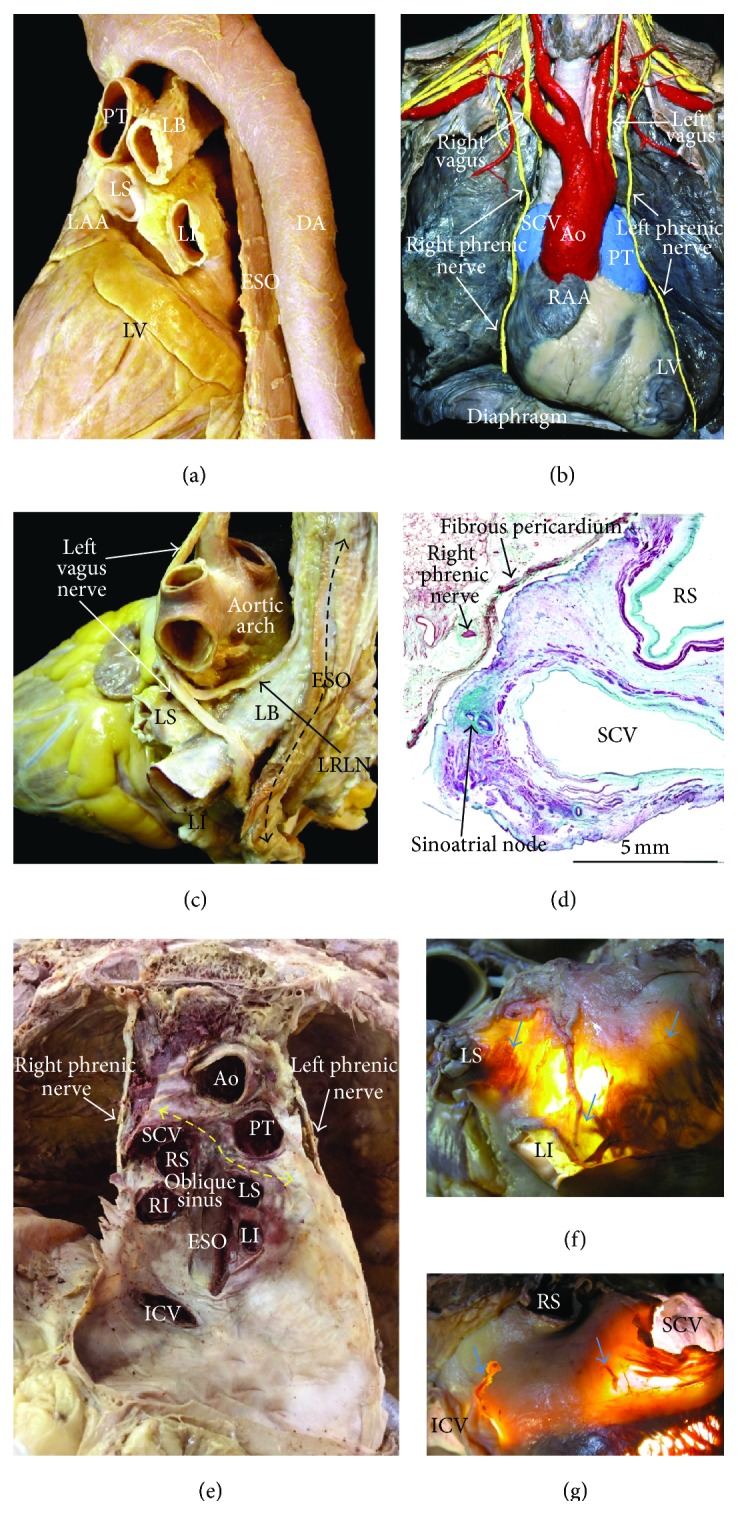
(a) Specimen viewed from a left posterolateral perspective to show the course of the esophagus and descending aorta relative to the left atrium. (b) This dissection of a cadaver viewed from the front shows the course of the right and left phrenic nerves. (c) Dissection of the left vagus nerve and its branch the left recurrent laryngeal which descends onto the roof of the left atrium. (d) Histological sections (Masson's trichrome stain) through the right superior pulmonary vein and superior cava vein. The right phrenic nerve is adherent to the fibrous pericardium. Note the short distance between the endocardium of these veins and the right phrenic nerve. (e) This dissection of a cadaver viewed from the front shows the transverse (double-headed yellow broken line) and oblique sinuses following removal of the heart. Note that we have opened a window on the oblique sinus to show the close anatomic relationship of the esophagus with the posterior left atrial wall. (f, g) Transillumination of the roof of the left atrium and posterior interatrial groove showing the acetylcholinesterase stained epicardial ganglionated nerves (blue arrows) extending to the superior surface of the left venoatrial junctions in (f) and intercaval region in (g). Ao = aorta, LB = left bronchus, DA = descending aorta, ESO = esophagus, ICV = inferior cava vein, LI = left inferior pulmonary vein, LRLN = left recurrent laryngeal nerve, LS = left superior pulmonary vein, LV = left ventricle, PT = pulmonary trunk, RAA = right atrial appendage, RI = right inferior pulmonary vein, RS = right superior pulmonary vein, and SCV = superior cava vein.

*(a) Esophagus*. Due to the close proximity of the esophagus to the posterior wall of the LA [58] (Figure 11), ablation procedures involving this region of the LA may cause esophageal damage and result in the formation of an atrial esophageal fistula [59]. The esophageal blood supply is located in the anterior wall of the esophagus, further increasing the risk caused by energy application in this region [58]. MDCT is a valuable imaging tool for showing the relationship of the atrial wall and the esophagus and descending aorta prior to the ablation procedure [60]. Peristalsis and dynamic movement of the esophagus during the procedure can result in discordance between the preprocedure and intraprocedure anatomy. Similarly, temperature probes might not necessarily be in the closest position toward the ablation catheter but might be buried inside the folding mucosa of the esophagus or along its posterior wall. Low readings may therefore give a wrong sense of security. Low ablation energy and as few ablations as necessary at the posterior wall seem to be the most careful way to avoid collateral damage.

*(b) Phrenic Nerves, Left Recurrent Laryngeal Nerve, and Cardiac Vagi*. The phrenic nerves lie along the lateral mediastinum and run from the thoracic inlet to the diaphragm [61, 62] (Figure 11). Phrenic nerve injury results from direct thermal injury, usually to the right phrenic nerve, which is located near the right superior PV and the SVC [61, 63] (Figure 9). Measurements made in cadavers show that the right phrenic nerve has a close relationship with the SVC (0.3 ± 0.5 mm) and the right superior pulmonary vein (2.1 ± 0.4 mm). Less frequently, ablation within the left atrial appendage and left lateral wall can result in left phrenic nerve damage [61, 62] (Figure 11). MDCT coronary angiography can demonstrate the left phrenic neurovascular bundle as it passes over the LV pericardium in 74% of the studies [64]. However, it is difficult to image the right phrenic nerve. In some high-quality images, the right phrenic nerve can be seen to be mildly enhancing the extrapericardial structure lateral to the SVC and anterior to the right superior pulmonary vein. In addition to the phrenic nerves, lesions along the roof of the atrium can damage the left recurrent laryngeal nerve, which courses below the aorta near the ligamentum arteriosum and then ascends (recurs) in the groove between the trachea and esophagus (Figure 11) providing innervation to the muscles of the larynx. A potential complication associated with left atrial ablation is recurrent laryngeal nerve injury leading to transient vocal cord paralysis [65]. Finally, the vagal plexus descends on the anterior surface of the esophagus and, therefore, behind the left atrium (Figure 11), and injury to this plexus can result in pyloric spasm and gastric hypomotility [66]. Our anatomical observation on normal cadavers revealed a mean distance between the bundles of the anterior esophageal plexus and posterior left atrial endocardium or venoatrial junctions of 4.1 ± 1.4 mm (range 2.5 to 6.5 mm) [67].

*(c) Ganglionated Plexus*. The heart is equipped with an intrinsic nervous system. The neural cell bodies of this system that are located in so-called ganglionated plexi found are mainly distributed on (1) the superior surface of the right atrium, (2) the superior surface of the left atrium, (3) the posterior surface of the right atrium, (4) the posterior medial surface of the left atrium (the latter two fuse medially where they extend anteriorly into the interatrial septum), and (5) the inferior and lateral aspect of the posterior left atrium and left PVs (Figure 11) [68, 69]. Endocardial ablation strategies that target those epicardial structures risk collateral damage, while epicardial ablation, for example, by minimally invasive techniques, at least allows for a more targeted approach. 

*(d) Transverse and Oblique Pericardial Sinus*. The pericardial access to the LA and PVs is limited by the fact that the pericardial sac folds around the heart. Access between the PVs is via the oblique sinus, which is formed by continuity between the reflections along the PVs and caval veins. It is a large cul-de-sac behind the LA (Figure 11). The access to the anterior part of the LA can be researched via the transverse sinus, lying between the back of the ascending aorta and pulmonary trunk bifurcation and the front of the atrial chambers (Figure 11).

## 5. Right and Left Ventricles

**Figure 12:**
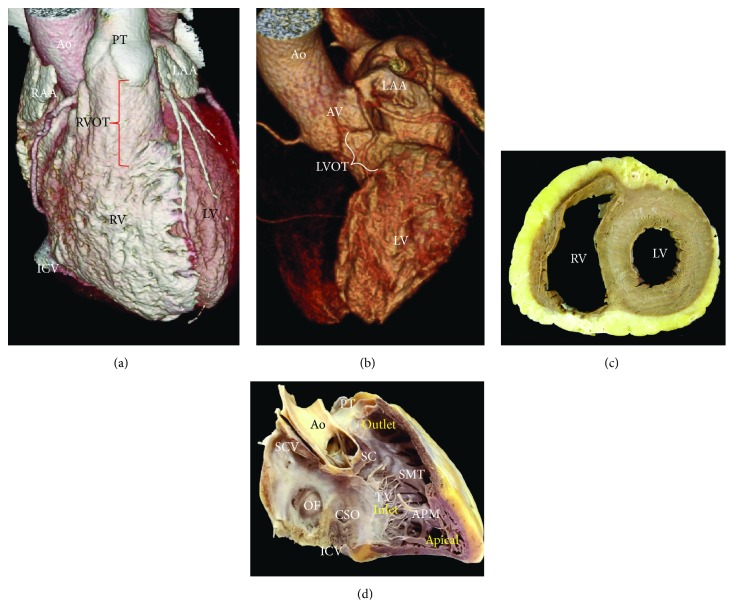
Right anterior views of volume rendered CT angiographies. (a) Right ventricle and (b) left ventricle. (c) The right ventricle is crescent-shaped in cross section. (d) This is a right lateral view showing the three components of the right ventricle and the characteristic muscle bundles as septomarginal trabeculation and supraventricular crest or ventriculoinfundibular fold. Ao = aorta, APM = anterior papillary muscle, AV = aortic valve, CSO = coronary sinus ostium, ICV = inferior cava vein, LAA = left atrial appendage, LV = left ventricle, LVOT = Left ventricle outflow tract, OF = oval fossa, PT = pulmonary trunk, RAA = right atrial appendage, RV = right ventricle, RVOT = right ventricle outflow tract, SC = supraventricular crest, SCV = superior cava vein, SMT = septomarginal trabeculation, and TV = tricuspid valve.

**Figure 13:**
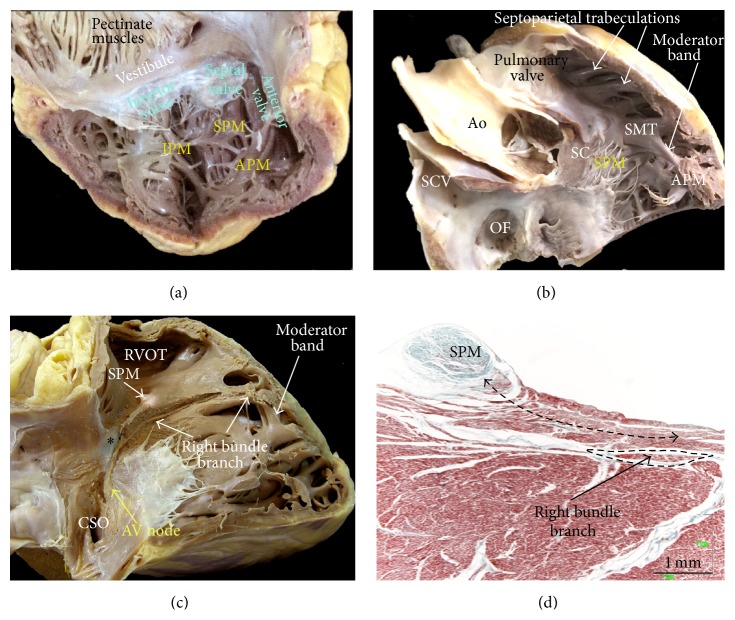
(a) The right side of the heart is opened to show the hinge of the tricuspid valve and the location of the right papillary muscles and leaflets of the tricuspid valve. (b) The right ventricle is opened from the front to show the septal papillary muscle, moderator band, and septoparietal trabeculations. (c) Window dissection of the right heart to show the AV node and right bundle branch crossing the central fibrous body (*∗*). (d) Histological section of the septal papillary muscle (double-headed black broken line). Note the close relationship with right bundle branch of His. Ao = aorta, APM = anterior papillary muscle, CSO = coronary sinus ostium, IPM = inferior papillary muscle, OF = oval fossa, RVOT = right ventricle outflow tract, SC = supraventricular crest, SCV = superior cava vein, SMT = septomarginal trabeculation, and SPM = septal papillary muscle.

The right ventricle (RV) is the most anteriorly situated cardiac chamber because it is located immediately behind the sternum. The RV is triangular in shape when viewed from the front and it curves over the left ventricle (LV) (Figure 12) [70]. In contrast, most of the LV is behind the RV with its outlet overlapping its inlet. This is because the central location of the aortic valve places the outflow tract in between the mitral valve (MV) and the ventricular septum (Figure 12). In cross section, the geometry of the RV is also influenced by the convexity of the ventricular septum (Figure 12). Both the RV and the LV have been described as having three components, the inlet (inflow tract), apical trabecular, and outlet (outflow tract) portions, but there are no discrete borders between adjacent portions (Figure 12). The inlet portion extends from the tricuspid annulus to the papillary muscles that anchor the tendinous cords and leaflets to the ventricular wall. The leaflets of the tricuspid valve can be distinguished as septal, anterosuperior, and inferior or posterior (Figure 13). The septal leaflet with its cords or medial papillary muscle inserting directly into the ventricular septum which is characteristic of the RV (Figure 10). Morphologically, the RV is distinguished from the LV by having coarser muscular trabeculations, a moderator band, and a lack of fibrous continuity between its inlet and outflow valves (Figures 12 and 13). The muscular trabeculations in the apical part of the RV are coarser than those in the LV [71]. One of the trabeculations is the moderator band, which is a bridge between the septomarginal trabeculation (SMT) or septal band to the anterior papillary muscle (Figures 12 and 13). The right bundle branch of His emerges from the membranous septum to descend subendocardially on the septomarginal trabeculation and the moderator band, which carries within it a major fascicle of the right bundle branch (Figure 13) [72].

The septomarginal trabeculation itself is a Y-shaped muscular band that is adherent to the septal surface. Between its limbs lies the infolding of the heart wall forming the ventricular roof, an area also known as the supraventricular crest that separates the inflow and outflow components of the RV and supports the pulmonary valve [73]. The septoparietal trabeculations take their origin from the anterior margin of the SMT and run round the free wall parts of the subpulmonary infundibulum (Figure 13) [74, 75]. These trabeculations show variable extension (between five and 22 trabeculations) and thickness (range 2–10 mm) along the right and left septoparietal wall of the RVOT.

**Figure 14:**
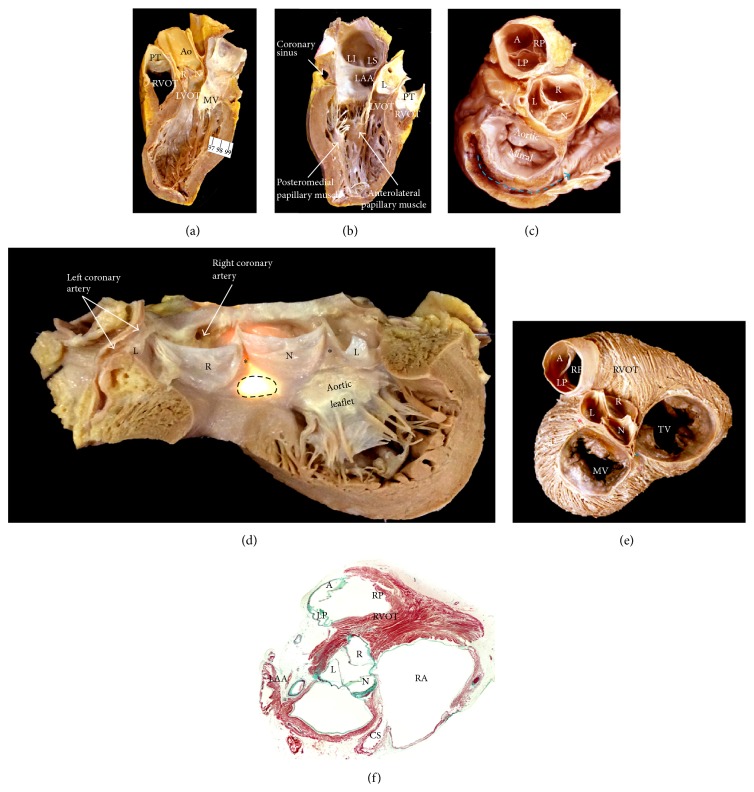
(a, b) These two halves of a heart are bisected longitudinally to show the three aortic sinuses and the papillary muscles of the mitral valve. Note that the muscular subpulmonary infundibulum of the right ventricular outflow tract abuts the right and left coronary sinuses. (c) The kidney-shaped vestibule of the mitral valve is shown from the atrial side. The commissure between the leaflets of the mitral valve and the fibrous continuity between the aortic leaflet of the mitral valve and left coronary and noncoronary of the aortic sinuses are shown. There is no muscular infundibulum in the left ventricle. Note the coronary sinus (blue broken line) next to the left atrioventricular groove surrounding the mitral valve from behind. (d) The aortic root has been opened through the left coronary aortic sinus showing the musculature in the depth of the sinus and the area of fibrous continuity between the leaflets of the aortic and mitral valves. It is photographed by transillumination from behind to show the three sinuses showing the left aortic sinus (L), the right aortic sinus (R), and the posterior or noncoronary aortic sinus (N). Two of the three interleaflet fibrous triangles are shown (*∗*). Note that the right fibrous trigone is continuous with the membranous septum, the two fibrous structures together forming the so-called central fibrous body (the brightest area by transillumination, black broken line). The specialized atrioventricular conduction axis penetrates through this fibrous area and left bundle branch. (e) Short-axis view from atria side, after removal of the epicardium and coronary vessels, shows the relationship of the aortic valve and the right (blue asterisk) and left (red asterisk) fibrous trigones. Pulmonary sinuses are named according to their relationship to the heart (nonattitudinal), including anterior (A), left posterior (LP), and right posterior (RP) pulmonary sinus. (f) Cross-histological section stained with Masson's trichrome through the left and the right atria. Note the anatomic relation of the right ventricular outflow tract with the subaortic outflow. Note that while all of the leaflets of the pulmonary valve are supported by infundibular musculature, only two of the leaflets of the aortic valve have muscular support. Ao = aorta, CS = coronary sinus, MV = mitral valve, LVOT = left ventricle outflow tract, PT = pulmonary trunk, RVOT = right ventricle outflow tract, and TV = tricuspid valve.

**Figure 15:**
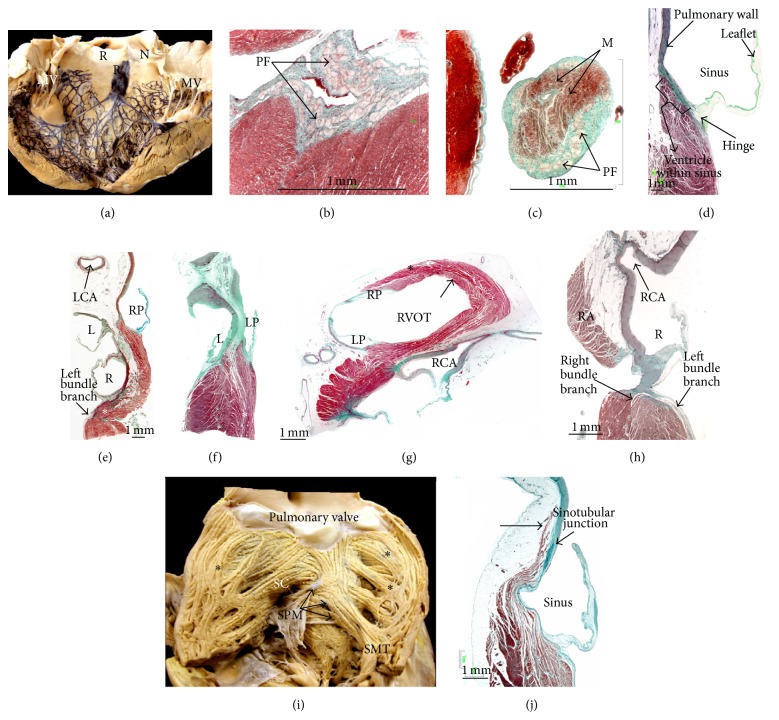
(a) This bovine heart shows the left bundle branch and Purkinje network after Indian ink injection. (b) Histological section stained with Masson's trichrome in a human heart showing Purkinje fibers at the level of the subendocardium of the left ventricle. (c) Histological section of a false tendon in a human heart which presents within Purkinje fibers, myocytes, and connective tissue in green colour. (d) Histological section of the pulmonary valve shows how the hinge of the valvar leaflet is attached to the ventricular myocardium well proximal to the anatomic ventriculoarterial junction. (e, f) Histologic sagittal sections of the right ventricular infundibulum, pulmonary valve root, left ventricle outflow tract, and aortic root. Note the differences in length of the right ventricular outflow tract infundibulum and in the contact area between both outflow tracts (red dotted lines) depending on the level of the section: at the right posterior pulmonary leaflet (e) or left posterior pulmonary leaflet (f). The subendocardial fibers in the infundibulum run longitudinally. At subendocardial levels of the left ventricular outflow tract, the orientation is mainly spiral and circumferential. Note that there are connections (asterisks) between myocytes in the contact area between both outflow tracts. (g) Histologic cross section of infundibulum of pulmonary valve stained with Masson's trichrome. In right ventricle, fibers orientation is different in infundibulum and at subendocardial (*∗*) and subepicardial (arrow) levels of pulmonary valve. (h) Histologic sagittal section of the aortic valve root at the level of right coronary leaflet. Note the relationship of the aortic valve with the conduction tissue and right atrium. (i) Endocardial view of right ventricular outflow tract (RVOT) is shown (d). Note that endocardial infundibular sleeve consists of septoparietal trabeculations (*∗*) arising from septomarginal trabeculation (SMT). Note crossing architecture pattern of myocardial strands between septomarginal trabeculation with septoparietal trabeculations and supraventricular crest below pulmonary valve. (j) Histological section of the pulmonary valve stained with Masson's trichrome shows the attachment of the pulmonary leaflet. Note the myocardial extension above the sinotubular junction (arrow). L = the left aortic sinus, LCA = left coronary artery, M = myocyte, MV = mitral valve, N = noncoronary aortic sinus, LP = left posterior pulmonary sinus, PF = Purkinje fiber, PT = pulmonary trunk, R = right aortic sinus, RA = right atrium, RCA = right coronary artery, RP = right posterior pulmonary sinus, RVOT = right ventricle outflow tract, SC = supraventricular crest, SCV = superior cava vein, SMT = septomarginal trabeculation, and SPM = septal papillary muscle.

The inlets also differ notably in the normal ventricles, as do the outlets [76] (Figure 14). The MV possesses two leaflets located anteriorly (aortic leaflet) and posteriorly (mural leaflet) but positioned obliquely within the LV and closing along a solitary zone of apposition. The mitral leaflets are attached via tendinous cords to two papillary muscles, the anterolateral (which is really superiorly and posteriorly located) and inferoseptal (which is positioned anteriorly) papillary muscles (Figure 14) [77]. Two-thirds of the MV are at the parietal atrioventricular junction (mural leaflet), whereas one-third is the span of fibrous continuity between the anterior leaflet and the aortic valve (Figure 14). The anterior leaflet of the MV is separated from the septum by the subaortic vestibule, having fibrous continuity with two of the leaflets of the aortic valve. The extremities of the fibrous continuity are the left and right fibrous trigones (Figure 14), the right forming part of the central fibrous body [78]. The landmark for the site of the atrioventricular bundle of His is the central fibrous body that the crescentic hinge lines of the right and noncoronary leaflet of the aortic valve (Figure 14). From here, the left bundle branch descends in the subendocardium and usually branches into three main fascicles that interconnect and further divide into finer Purkinje network (Figure 15). Occasionally, fine muscular strands or false tendons extend between the septum and the left papillary muscles or the parietal wall [79]. Often, they carry the distal ramifications of the left bundle branch. Approximately 10% to 20% of sustained monomorphic idiopathic ventricular tachycardia occurs in apparently structurally normal hearts [80]. Often they are focal in origin, relating to the Purkinje fiber network. Some data suggest origin from a false tendon or fibromuscular band (Figure 15). Other sources have been mapped to the fascicles of the left bundle branch, the inferior and paraseptal region of the mitral annulus, and the left ventricular outflow tract, which may require the nadirs of the aortic sinuses to be targeted.

## 6. Anatomy of the Outflow Tracts: Implication for Ablation of Ventricular Tachycardias

The right ventricular outflow tract (RVOT) passes superiorly and cephalad over the left ventricular outflow tract (LVOT), which is directed rightward, resulting in a crossover relationship between the two tracts, in fact most of the RVOT is to the left of the LVOT (Figures 14 and 15), and the left main coronary artery is closer to the posterior RVOT than it is to the depths of the left coronary leaflet. The configuration of the semilunar leaflets of the pulmonary valve results in the crescentic hinge lines crossing the anatomical junction between ventricular musculature and arterial wall, enclosing within the nadirs of the valvar sinuses small areas of myocardium (Figure 15). The plane of the pulmonary valve is higher and nearly horizontal, whereas that of the aortic valve is tilted lower and at an angle of at least 45° from the median plane. The difference in levels between the two sets of arterial valves may be exaggerated by the length of infundibulum. The myocardium of the posterior RVOT is essentially in continuity with the LVOT and the adjacent anterior interventricular septum (Figures 14 and 15). The more distal posterior RVOT myocardium is immediately adjacent to the aortic leaflets (right coronary leaflet and part of the left coronary leaflet). The myocardium is relatively thin in the rightward, anterior, and subpulmonary valve portions of the RVOT, whereas the posterior infundibular part that is adherent to the anterior LVOT as a continuation cephalad of interventricular septum is the thickest. Because of the leftward proximal-to-distal course of the RVOT, the most rightward portion of the right outflow tract is the bundle of His region, and the most leftward portion is the supravalvar myocardium above the anterior leaflet of the pulmonary valve.

The LVOT is much more reduced in size because of the fibrous continuity between two of the leaflets of the aortic valve and the aortic leaflet of the MV (Figure 14). Therefore, in the LV there is no muscular separation between inflow and outflow tracts [76]. Although the two ventricular outlets have important differences in their structure, they also have one feature in common, namely, the semilunar attachment of their leaflets. Because of the semilunar shape of the pulmonary leaflets this valve does not have ring-like annulus [74, 75]. The semilunar hinges of the arterial valve leaflets extend proximally beyond the anatomic ventriculoarterial junction such that crescents of myocardium are incorporated into the bases of all three valvar sinuses of the pulmonary valve and into two of the three aortic sinuses of Valsalva (Figure 14). The coronary orifices are usually located in the sinuses immediately below the level of the sinutubular junction rather than toward the nadirs.

Premature ventricular contractions, ventricular tachycardias (VTs), and initiating beats for ventricular fibrillation have all been localized at the level of the right and left ventricular outflow tracts (RVOT and LVOT) [80, 81]. Absence of structural heart disease is the rule with these arrhythmias. The majority of RVOT tachycardias originate in the superior, septal, and anterior aspects of the infundibulum just underneath the pulmonary valve [82]. Owing to the spatial relationship of the subpulmonary infundibulum and the left ventricular outlet, the foci may be ablated from within the part of the RV outlet that overlies the adjacent aortic sinuses, and vice versa [81] (Figure 15). The noncoronary aortic sinus, immediately adjacent to the paraseptal region of the left and right atria and close to the superior atrioventricular junction, may be used to map and ablate focal atrial tachycardia in the vicinity of the His bundle (Figure 15). We observed in histological sections the existence of myocardial extensions or myocardial remnants on the epicardial aspect above the sinotubular junction in 20% of human specimens, showing continuity with the myocardium of the RVOT (Figure 15). These extensions could justify the existence of idiopathic supravalvular tachycardia. Although not histologically documented, published case examples of myocardial extensions extending to the vicinity or possibly into the coronary artery ostia have been reported [83–85]. The ultrastructure of these myocardial extensions is not as well worked out as the pulmonary veins. It is unknown whether this myocardium is similar to or different to the adjacent atrial and ventricular myocardium, whether fibrosis is present, and whether or not cell-to-cell junctions and connections between the arterial smooth muscle and the myocardium exist.

## 7. Conclusions

Understanding the image detail of the heart obtained with echocardiography and CT has an imperative role in outlining and anatomically delineating the cardiac landmarks related to the conduction system. Learning classic anatomy of the heart provided by cadaveric specimens is the prerequisite to understand what imaging delivers. Since anatomic variation of the cardiac conduction system landmarks and associated structures is common, it is also crucial to learn more about these normal variants, especially prior to interventional procedures.
